# Supplementary material for: Beating Adolescent Self-Harm (BASH): a randomised controlled trial comparing usual care versus usual care plus a smartphone self-harm prevention app (BlueIce) in young adolescents aged 12–17 who self-harm: study protocol
Source: BMJ Open. 2021 Nov 23;11(11):e049859. doi: 10.1136/bmjopen-2021-049859 (PMC8611450; doi:10.1136/bmjopen-2021-049859)
Supplement: Author's manuscript [file bmjopen-2021-049859.draft_revisions.pdf]

BMJ Open is committed to open peer review. As part of this commitment we make the peer review history of every article we publish publicly available.

When an article is published we post the peer reviewers' comments and the authors' responses online. We also post the versions of the paper that were used during peer review. These are the versions that the peer review comments apply to.

The versions of the paper that follow are the versions that were submitted during the peer review process. They are not the versions of record or the final published versions. They should not be cited or distributed as the published version of this manuscript.

BMJ Open is an open access journal and the full, final, typeset and author-corrected version of record of the manuscript is available on our site with no access controls, subscription charges or pay-per-view fees (http://bmjopen.bmj.com).

If you have any questions on BMJ Open's open peer review process please email info.bmjopen@bmj.com

BMJ Open

Beating Adolescent Self harm (BASH). A randomised controlled trial comparing usual care versus usual care plus a smartphone self-harm prevention app (BlueIce) in young adolescents aged 12-17 who self-harm: study protocol.

Journal:	*BMJ Open*
Manuscript ID	bmjopen-2021-049859
Article Type:	Protocol
Date Submitted by the Author:	04-Feb-2021
Complete List of Authors:	Greenhalgh, Isobel; NHS, Child and Adolescent Mental Health Services<br>Tingley, Jessica; NHS, Child and Adolescent Mental Health Services<br>Stallard , P; NHS<br>Taylor, Gordon; University of Exeter, College of Medicine and Health<br>Medina-Lara, Antonieta; University of Exeter Medical School, Health Economics Group<br>Rhodes, Shelley; University of Exeter, Medical School
Keywords:	MENTAL HEALTH, Suicide & self-harm < PSYCHIATRY, Telemedicine < BIOTECHNOLOGY & BIOINFORMATICS

SCHOLARONE™
Manuscripts

I, the Submitting Author has the right to grant and does grant on behalf of all authors of the Work (as defined in the below author licence), an exclusive licence and/or a non-exclusive licence for contributions from authors who are: i) UK Crown employees; ii) where BMJ has agreed a CC-BY licence shall apply, and/or iii) in accordance with the terms applicable for US Federal Government officers or employees acting as part of their official duties; on a worldwide, perpetual, irrevocable, royalty-free basis to BMJ Publishing Group Ltd ("BMJ") its licensees and where the relevant Journal is co-owned by BMJ to the co-owners of the Journal, to publish the Work in this journal and any other BMJ products and to exploit all rights, as set out in our licence.

The Submitting Author accepts and understands that any supply made under these terms is made by BMJ to the Submitting Author unless you are acting as an employee on behalf of your employer or a postgraduate student of an affiliated institution which is paying any applicable article publishing charge ("APC") for Open Access articles. Where the Submitting Author wishes to make the Work available on an Open Access basis (and intends to pay the relevant APC), the terms of reuse of such Open Access shall be governed by a Creative Commons licence – details of these licences and which Creative Commons licence will apply to this Work are set out in our licence referred to above.

Other than as permitted in any relevant BMJ Author's Self Archiving Policies, I confirm this Work has not been accepted for publication elsewhere, is not being considered for publication elsewhere and does not duplicate material already published. I confirm all authors consent to publication of this Work and authorise the granting of this licence.

For peer review only

Beating Adolescent Self harm (BASH). A randomised controlled trial comparing usual care versus usual care plus a smartphone self-harm prevention app (BlueIce) in young adolescents aged 12-17 who self-harm: study protocol.

Greenhalgh I[1], Tingley J[1], Taylor G[2], Medina-Lara A[3], Rhodes S[2], & Stallard P[1].

Author Information

1. Child and Adolescent Mental Health Service (CAMHS), NHS, Keynsham, UK
2. College of Medicine and Health, University of Exeter, Exeter, UK
3. Clinical Trials Unit, University of Exeter, Exeter, UK

Full name, postal address and email of the corresponding author:

Paul Stallard, 6.10 Wessex House, Department of Health, University of Bath, BA2 7AY, UK. P.stallard@bath.ac.uk

Word count: 4493.

For peer review only

ABSTRACT

Introduction

A mobile app, BlueIce, was co-designed with young people with a history of self-harm with the aim of providing more accessible evidence-based support at times of distress. A preliminary evaluation found that BlueIce was acceptable, safe, and used by young people, and helped to reduce self-harm. The current study aims to determine the effectiveness and cost-effectiveness of adding BlueIce to usual specialist child and adolescent mental health service (CAMHS) interventions on self-harm and mental health.

Methods and analysis

Single blind, randomised controlled trial comparing usual CAMHS care with usual care plus BlueIce. 138 adolescents aged 12-17 with current or a history of self-harm will be recruited through the Oxford Health NHS Foundation Trust via their CAMHS clinician. Assessments of self-harm, mood, anxiety, hopelessness, general behaviour, sleep, impact on everyday life, health related quality of life and health care resource utilisation will be collected at baseline, 12 weeks and 6 months. Post-use interviews with young people will be undertaken at 12 weeks to determine the acceptability, safety and usability of BlueIce.

Ethics and dissemination

The project has been reviewed and approved by the South Central – Oxford B NHS Research Ethics Committee (19/SC/0212) and approved by the HRA and Health and Care Research Wales. The findings of this study will be disseminated through submission to high impact open access publications and through presentations at relevant academic and clinical conferences. Results will be made available to all participants after the completion of the study.

Trial registration: ISRCTN registry, registration number ISRCTN 10541045. Registered on 22/07/2019

Strengths and limitations of this study

- This is the first adequately powered randomised controlled trial of a digital self-help app for young people aged 12-17 years who self-harm.
- This study will add to the evidence-base and will document the effectiveness and cost-effectiveness of adding a self-help app to usual care.
- We have minimized the possibility of contamination between the trial arms as BlueIce is a prescribed app with a single-use download code.
- Researchers are blind to participants
- This study is investigating the effectiveness of BlueIce as a prescribed app (used alongside specialist mental health care) and we will not determine the efficacy of BlueIce as a standalone intervention.

For peer review only

For peer review only - http://bmjopen.bmj.com/site/about/guidelines.xhtml

Keywords

BlueIce, Self-Harm, Children, Adolescents, Mobile App. CAMHS, Mental Health, (m)Health

For peer review only

INTRODUCTION

Background

Self-harm is defined as the deliberate act of causing damage to one's body, for example through self-poisoning or self-cutting [1,2]. It is very prevalent, with around 17.2% of adolescents and 13.4% of young adults being estimated to self-harm [3]. Rates of self-harm are more likely to reduce in young adults compared with younger adolescents [4]. Most self-harm occurs in secret with comparatively few episodes resulting in hospital presentations [5-7]. While suicide is less prevalent amongst younger adolescents, it is the third most common cause of death in young people [8]. Self-harm has consistently been cited a risk factor for later suicide attempts [9].

Research has identified risk factors for self-harm, including: negative and stressful life events such as childhood maltreatment; self-harm or attempted suicide of a family member; drug and alcohol abuse; psychological factors such as feelings of hopelessness, perfectionist traits, emotional dysregulation, or diagnoses of psychological disorders; lower sociodemographic status; and being female [7,10-12]. Protective factors which may help reduce or prevent self-harm include better access to social support, higher levels of self-esteem and receiving support from parents, as well as being able to re-evaluate one's own thoughts and beliefs [4].

Evidence-based interventions for self-harm in children and adolescents are scarce. Few studies have evaluated cognitive behaviour therapy (CBT) or problem-solving approaches [13,14]. Family therapy does not appear effective [15]. There is some support for dialectical behaviour therapy (DBT) [16-19], and for mentalization-based treatment (MBT) [20]. However, a Cochrane review concluded that there is not much evidence on which to draw conclusions on the effects of interventions for adolescent self-harm and recommended that therapeutic assessment, metallization, dialectical behaviour and cognitive behaviour therapy warrant further evaluation [13]. With recent advance in technology the use of digital interventions to aid the delivery of mental health interventions has become more widespread. The NHS is encouraging the use of technology in order to improve access to, and the availability of, support and interventions, and to reduce demands on mental health services [21].

There are currently over 15,000 mobile (m)health apps available worldwide, with around 5,000 of these targeted at mental health [22]. Apps offer an accessible way of delivering and supporting mental health interventions for young people since over 80% of 12-15-year olds own a smartphone [23]. Alongside improving accessibility to support, mHealth apps are available 24/7, provide a means for symptom monitoring between face-to-face meetings, may be a preferable method of accessing support for some young people, and provide a route around barriers such as stigma. mHealth apps may also help to lessen the demand on healthcare resources [22,24,25]. However, the evidence base surrounding healthcare apps is severely lacking, with research unable to keep pace with the speed of their development [24]. Therefore, the safety, efficacy and acceptability of the majority of apps is unknown.

In response to the above, a self-help app, BlueIce, was co-developed with young people with a lived experience of self-harm [26]. It is designed to support self-management of distress and reduce self-harm behaviours. BlueIce has 24/7 accessibility and is a prescribed app to

For peer review only - http://bmjopen.bmj.com/site/about/guidelines.xhtml

For peer review only

be used alongside face-to-face Child and Adolescent Mental Health Services (CAMHS) [27]. BlueIce is password protected and all data is stored locally on the phone [26]. It meets all minimum standards required for NHS accredited apps [28].

BlueIce utilises evidence based approaches of CBT and DBT and includes 3 sections:1) mood diary; 2) mood lifting section; and 3) emergency contacts. Upon entering BlueIce, young people rate their mood and, if this is rated as low, they are routed to the mood-lifting section, which is also accessible from the main menu. The mood lifting section contains 8 personalised activities to enhance mood, including: a photo and music library, physical activities, mood-changing activities, calming exercises, a thought diary, DBT skills (pros and cons, self-soothe and ice-dive), and phone numbers of key contacts they can call or message. If young people rate their mood as low following the use of the mood lifting section, they are routed to the final section which provides emergency contact numbers they can call for support [27].

An initial open study to determine safety, acceptability and usability found that 73% (19/26) of BlueIce users reported a reduction or cessation of self-harm at the 12-week follow-up [27]. No participants reported an increase in self-harm during the study. Post-use reductions in symptoms of anxiety and depression were also reported with 88% (29/33) of users electing to keep the app at the end of the study [27].

Study Objective

The aims of this trial are to determine the effectiveness, cost- effectiveness and acceptability of adding BlueIce to usual face-to-face specialist mental health care in the reduction of self-harm in adolescents.

For peer review only - http://bmjopen.bmj.com/site/about/guidelines.xhtml

METHODS

Trial design

This is a two arm, single blind, randomised controlled trial (RCT) comparing the addition of the BlueIce self-help app to usual face-to-face specialist mental health care (UC+BI) with usual specialist mental health care (UC).

Setting and participants

Participants will be recruited through CAMHS provided by Oxford Health NHS Foundation Trust, covering North-East Somerset, Bath, Swindon, Wiltshire, Buckinghamshire and Oxfordshire.

Young people will be eligible if they: (1) are receiving treatment from CAMHS at the time of referral; (2) have self-harmed at least twice in the last 12 months; and (3) are aged between 12 and 17 years.

Exclusion criteria are: (i) a diagnosis of psychosis; (ii) a significant learning disability which would interfere with the young person's ability to use the app; (iii) young people with active suicidal plans, or (iv) safeguarding concerns where the young person has suffered abuse within the last 6 months or is the subject of a safeguarding investigation.

Recruitment

Clinicians across Oxford Health NHS Foundation Trust will be invited to identify eligible young people who are open to CAMHS. Clinicians will discuss the study and provide interested young people, and if under 16 their parents/carers, with a project information sheet. If interested in participating, their details will be forwarded to the research team.

Consent

Researchers will meet with the young people, and if appropriate their parents/carers, to discuss the project. If the young person wants to take part, the researcher will obtain consent. If under 16 years old, the young person will be asked to provide assent while their parent or legal guardian will be asked for consent. Those aged 16 years or older will be able to provide their own signed consent. During the COVID pandemic the consent and assessment process will be undertaken remotely (i.e. online or via telephone) in order to maintain the safety of participants and the research team.

Randomisation

Computer generated randomisation will be independently undertaken by Exeter Clinical Trials Unit. Participants will be randomised in a 1:1 ratio to either usual specialist mental health care (UC) or usual specialist mental health care plus the BlueIce self-help app (UC+BI). Participants will be randomised using REDCap software minimising for gender, age

For peer review only - http://bmjopen.bmj.com/site/about/guidelines.xhtml

(over or under 16), self-harm frequency in last 4 weeks (0-2 or ≥3), and severity of depression (Mood and Feelings Questionnaire above or below 27, the cut off for severe depression). Following guidance from our participation group, those allocated to usual care will be provided with BlueIce at their final, six-month assessment.

Blinding

Due to the nature of the intervention it is not feasible to blind participants to allocation. The research team are aware of the need to maintain equipoise and to present the two interventions to referring clinicians and participants in a balanced way. Researchers will remain blind to treatment arm and, if this is inadvertently broken, subsequent assessments will be conducted by another member of the research team blind to participant status.

Interventions

Usual Care (UC): Young people will receive individual mental health interventions from specialist CAMHS clinicians. This will be either face-to-face, or, due to COVID, a remotely delivered telephone or video intervention. The nature, content and duration of this will be captured by the health care resource questionnaire.

Usual care plus BlueIce (UC+BI): In addition to usual care, young people will also receive access to the self-help BlueIce app. BlueIce is an application for android and apple smartphones. It contains a mood diary, personalised toolbox of mood lifting strategies that are available to the young person 24/7 and automatic routing to emergency contact numbers. (i). *Mood diary*: The young person is able to monitor their mood each day. For each mood rating the young person has the option of adding a note to record any particular reason why they might be feeling as they do. Their rating and notes are saved in a calendar which the young person and therapist can review to look for changes and patterns over time. (ii). *Mood lifting*: If the young person rates their mood as low, they will automatically be routed to the mood lifting section. Alternatively, if at any time the young person would like to access this section they can do so directly from the main menu. This section contains a menu of mood lifting activities personalised according to the interests of the young person. The activities are designed to counter the common reasons why young people self-harm (to punish themselves, emotional relief, feeling hopeless) and draws on common methods used in cognitive behaviour therapy (CBT) and dialectical behaviour therapy (DBT). The mood lifting section includes 8 activities: 1). Photo library: the young person can upload and save photographs, inspirational quotes and pictures that are associated with happy memories or which might make them feel good. These can be reviewed when low to help the young person remember the positive things in their life: 2). Music library: a music player is included where the young person can upload and store music they enjoy, and which has a positive effect on how they feel. This playlist can be readily accessed when the young person is low as a way of improving their mood: 3). Physical activities: the young person can identify physical activities they enjoy such as sporting activities (e.g. going for a run or riding a bike) or other aerobic activities such as walking the dog. The young person can access their personalised list when low and be reminded about what they can do to get active to improve their mood: 4). Mood changing activities: BlueIce includes a section of activities that make the young person feel good. These could be things like making a cake, watching an episode

For peer review only

of a favourite TV series, reading a book, playing with a pet. These provide the young person with a prompt list of activities they can use to change their mood when feeling down: 5). Relaxation and mindfulness exercises: audio-recorded instructions for a 10-minute mindfulness session, calming visualisation and a quick controlled breathing exercise (4-7-8 breathing) are included. These can be used to help the young person manage any unpleasant emotions or distressing thoughts: 6). Identification of negative thoughts: this section includes a thought diary where the young person can record any troubling thoughts that are racing through their head. These can be directly typed into BlueIce where they are saved and can be reviewed at a later date. This allows identification of any particular themes that could be addressed during face-to-face work with their clinician: 7). Ride it out: this section draws on ideas from DBT and helps the young person to tolerate their distress. This includes instructions for an ice dive, a sensory toolbox and a “pros and cons” balance sheet for self-harming: 8). Call a friend: the final section contains the phone numbers of 3-5 people who the young person could contact if they were feeling low and in danger of self-harming. These would be people who make them feel happy and those they could talk with about how they are feeling. This section prompts the young person to reach out to others. (iii) *Emergency contacts*. After accessing the mood lifter, the young person is asked to re-rate their mood. If they are still low and feeling that they might harm themselves, they will be routed through a series of questions to three emergency contact numbers. The young person can select one of these options to automatically call/text emergency support.

Patient and public involvement

BlueIce was co-designed and produced with young people with a lived experience of self-harm. They were involved in exploring the concept (would an app be helpful?), what an app should look like (examples of apps liked and used), the design (font, colours, flow) and content (evidence-based and ideas young people found helpful).

In this study, two young people will be recruited to join our Study Steering Committee (SSC). We plan participant workshops to develop study resources, to advise on recruitment and retention issues, to discuss study findings, identify key messages, prepare understandable research summaries in different formats and identify issues/implications for future research. We intend to involve young people in events disseminating the findings of the study.

Assessment schedule

Data will be collected at: (i) baseline; (ii) post-intervention (12 weeks); and, (iii) follow-up (6 months after randomisation). Data will be collected by Research Assistants, blind to treatment allocation. Participants will be given a £20 voucher after completing the final assessment.

Baseline: Standardised self-report measures of self-harm, depression, anxiety, hopelessness, general behaviour, impact on everyday life, sleep and health-related quality of life and resource use health care questionnaire. These will be complemented with case-note review to detail resource use i.e. accident and emergency department attendances, out of hours contacts, primary and secondary care attendances following incidents of self-harm in the preceding 6 months.

For peer review only - http://bmjopen.bmj.com/site/about/guidelines.xhtml

Post-intervention (12 weeks): Standardised self-report measures of self-harm, depression, anxiety, hopelessness, general behaviour, impact on everyday life, sleep and health-related quality of life and the resource use health care questionnaire will be repeated (baseline -12 weeks). Case note review will be repeated (baseline to 12 weeks) and the type and total hours of direct and indirect CAMHS intervention provided from randomisation to 12 weeks detailed. Those in UC + BI will complete a semi-structured interview detailing their use, experience of, and satisfaction with BlueIce.

Follow-up (6 months after randomisation): Standardised self-report measures of self-harm, depression, anxiety, hopelessness, general behaviour, impact on everyday life, sleep and health-related quality of life. Case note review will be repeated (12 weeks to 6 months) and the type and total hours of direct and indirect CAMHS intervention provided from 12 weeks to 6 months detailed.

Outcome measures

Primary Outcome: Our assessment of self-harm will consist of three parts: A). a brief interview; B). completion of the Risk Taking and Self-Harm Inventory for Adolescents (RTSHIA) [29] and C). the provision of support and advice.

Part A: Interview. Young people will be asked "have you ever hurt yourself on purpose in any way (e.g. by taking an overdose of pills or by cutting yourself) over the past 3 months?" which was taken from the Avon Longitudinal Study of Parents and Children (ALSPAC) (http://www.alspac.bris.ac.uk) [30]. Young people are asked to consider the last 6 months at baseline, and the last 3 months at follow-ups. Those who answer yes will be asked further questions about frequency, method, reason for self-harming, whether they sought medical help and suicidal intent.

Part B: RTSHIA. Our primary outcome is self-reported self-harm assessed by the self-harm inventory of the RTSHIA. The RTSHIA was developed in the UK for use with adolescents (aged 11-19 years) [20,29]. The self-harm inventory consists of 18 items and assesses the presence and frequency of a range of intentional self-injuries (e.g. cutting, burning, self-hitting, self-poisoning). The frequency of each item is rated on a 4-point scale (never, once, more than once, many times) over a defined period. At baseline, the young person is asked to consider the 6 months before their initial assessment. For the 12 week and 6-month assessment the young person considers the 3-month period since their last assessment. Each item is then scored (0,1,2,3) and totalled to provide a current self-harm score. The RTSHIA has good reliability and validity [20,29]. We will analyse total scores and use this information to categorise changes in self-harm from baseline to 12 weeks and 6 months as reduced/stopped vs same/increased.

Part C: Support and Advice. At the end of the assessment young people will be given a list of contacts they can call if they are feeling worried about themselves. These include NHS 111, Childline and the Samaritans.

Secondary outcomes: The Mood and Feelings Questionnaire (MFQ) [31], is a self-report questionnaire for depression recommended by NICE consisting of 33 items rated as either "true" (scores 2), "sometimes true" (scores 1) or not true (scores 0). The MFQ has high

For peer review only - http://bmjopen.bmj.com/site/about/guidelines.xhtml

criterion validity and correlates well with other measures of depression [31]. A total score of 27 and above is associated with major depression, 20 with mild depression and 16 with no mood disorder. The Hopelessness Scale for children, adapted from Beck's Hopelessness Scale [32,33], consists of 17 true–false items measuring hopelessness and negative expectations for the future. Items endorsed as "true" are summed, with higher scores indicating greater hopelessness. The Hopelessness Scale for children has been widely used within adolescent samples and has consistently demonstrated strong psychometric properties [32,33]. The Revised Child Anxiety and Depression Scale (RCADS) [34] is a 47-item questionnaire with items corresponding to DSM-IV criteria for anxiety in the areas of social phobia, separation anxiety, obsessive compulsive disorder, panic disorder, generalised anxiety disorder and for major depressive disorder. Each item is rated on a 4-point Likert scale of frequency (never 0; sometimes 1; often 2; always 3) which are summed to produce sub-scale and total anxiety scores. The Strengths and Difficulties Questionnaire (SDQ) [35] is a widely used behavioural screening questionnaire consisting of 25 items assessing emotional symptoms, conduct problems, hyperactivity and/or inattention, peer relationship problems, and pro-social behaviour. Each item is rated as not true (0), somewhat true (1) or certainly true (2). A total difficulties score is calculated by summing scores from all subscales except the pro-social. In addition, an impact supplement assesses the degree of distress created by the child's difficulties and the degree to which they interfere with home life, friendships, classroom leaning and leisure activities. The five items are summed to produce a total 'impact on everyday life' score which ranges from 0-10. The Sleep Condition Indictor (SCI) [36] is an eight item self-report measure assessing sleep and impact on daytime functioning over the past month on a 5-point scale. Item scores are summed to produce a total score ranging from 0-32. The SCI is an internally consistent ($\alpha = .86$) measure with a clinical cut-off <17 correctly identifying 89% of those with probable DSM-5 insomnia disorder [36].

Qualitative evaluation: We will use the semi-structured interview developed in our initial study [37] to assess participant's experience of BlueIce including use, ratings of satisfaction, helpfulness, ease of use and whether they would recommend it to a friend. In addition, we will assess the degree to which BlueIce was used and which parts of the app were used most often. This will only be completed by UC+BI at 12 weeks.

Economic Analysis: A cost-effectiveness analysis will be undertaken alongside the RCT to estimate the incremental cost-effectiveness of usual care + BlueIce (UC+BI) compared to usual care (UC) i.e. incremental cost per unit of health outcome (primary outcome) and Quality Adjusted Life Years (QALYs) using the NHS and social care perspective. In order to estimate QALYs we will use the Child Health Utility 9D (CHU-9D). This preference based generic HRQoL measure is designed specifically for use in the economic evaluation of health care interventions in young people [38]. The CHU-9D contains nine dimensions (worried, sad, pain, tired, annoyed, schoolwork/homework, sleep, daily routine and activities), each with five levels of functioning rated for "today". The CHU-9D has been validated for self-completion by young people (aged 7–17 years) [39] and with child and adolescent mental health services [40]. Resource use and cost data will be collected from the participants recruited in the RCT using a resource use questionnaire. Data on self-reported resource use will be compared with the self-harm assessment data that are recorded in clinical case notes (CareNotes). Clinical records will be reviewed for accident

For peer review only

For peer review only - http://bmjopen.bmj.com/site/about/guidelines.xhtml

and emergency attendances, out of hours contacts, or primary and secondary care attendances following self-harm over two periods (6 months to baseline; baseline to 6 months). We will also quantify the number of face-to-face appointments and total number of hours of CAMHS input provided from baseline to 6 months. Incremental costs will be combined with data on effectiveness/health outcomes. National unit costs will be obtained from available sources including PSSRU (https://www.pssru.ac.uk/) and from the National NHS cost collection (https://www.england.nhs.uk/national-cost-collection/). Analyses will follow good practice for conducting economic evaluations in health technology assessment [41] and findings will be reported using the CHEERS guidelines [42]. Results will include disaggregated data, as well as synthesis of cost and outcome data, and will include presentation of cost-effectiveness plane [43], cost-effectiveness acceptability curves [44], and detailed consideration of the broader impacts of the results reported. Robustness will be assessed through sensitivity analyses. Multiple imputation will be used to “fill-in” missing cost and outcome data, making the assumption that the data are missing at random [45]. If the young person is under 16, we will also ask their parent or guardian to complete both these questionnaires.

Sample size

A 3-point difference on our primary outcome (RTSHIA) between treatment groups represents a clinically important difference [29]. However, we propose to adopt a more conservative approach and will power the study to detect a moderate effect representing a 2-point difference. With a SD of 3.6, 90% power, alpha set at 0.05, we will require 69 participants per group.

Planned analysis

Statistical analysis

Our primary analysis will be at the end of the 12-week follow up of the last recruited participant. A detailed Statistical Analysis Plan (SAP) will be developed by the trial statistician in consultation with the project management group and agreed with the SSC before database lock. We will follow the CONSORT extension for reporting randomised controlled trials and will follow recommended guidelines for analysis of our data [46]. Our primary analysis at 12-weeks will be analysed on an intention to treat principle. Although we are not expecting a significant amount of missing data at 12 weeks, the impact of missing data will be assessed by comparing baseline covariates for missing and non-missing cases. In the event that there is evidence of bias being introduced into the analysis then further consideration, including but not limited to multiple imputation, will be given regarding how to address this.

Descriptive statistics will summarise baseline characteristics for each arm and patterns of missing follow-up data will be explored. We will also undertake a per protocol analysis of our primary outcome, total scores on the RTSHIA. Regression analysis adjusting for baseline minimisation variables of age, gender, mood and self-harm frequency will be undertaken. We will conduct sensitivity analyses in which we adjust for prognostic variables for which there is a baseline imbalance between intervention arms. Further sensitivity analyses will use multiple imputation to deal with missing data.

For peer review only - http://bmjopen.bmj.com/site/about/guidelines.xhtml

Similar regression analyses will be conducted for secondary outcomes (linear regression for numerical outcomes and logistic regression for binary outcomes). All secondary outcome measures will be compared between the groups and will include summary statistics and confidence intervals for measures of effect size.

Analysis of the 6-month data will be included in a repeated measures analysis to investigate the maintenance of any effect seen at 12 weeks. Analysis of the 6-month follow-up data will be undertaken using a repeated measures analysis of variance with both the 12 weeks and 6-month data being included and adjusted for the baseline. The analysis will also be adjusted for the baseline minimisation variables: age, gender, mood and frequency of self-harm as proposed for the primary analysis at 12 weeks.

Confidentiality

Each participant in the study will be assigned an ID code to keep data anonymous. All information collected will be linked to the specific ID to maintain anonymity. Any confidential data about the participants will be stored on a secure server in a password protected folder, which is only accessible to the study researchers. Furthermore, upon publication no ID codes or personal information will be available.

All information provided will be confidential unless a researcher is concerned that the young person or another is in immediate danger. If the researcher is concerned, they will contact the study lead, who may contact the young person’s lead clinician with the disclosed information.

Adverse event reporting and harms

For the purposes of this trial, adverse events are defined as increases in extent of self-harm or suicidal ideation regardless of whether they are casually related to the trial procedures. Serious adverse events are those requiring hospital admission. All adverse and serious adverse events will be reported to the project leader and will be reviewed by the Study Steering Committee

Referring clinicians will be requested to inform study researchers should a young person experience an adverse event during their participation in the study. At both follow-up visits, the young person (and parent if under 16) will be asked if any adverse events have occurred and if they think the study is having any negative effects on their mental health and self-harm frequency.

Ethics and dissemination

Favorable ethical opinion for the research was obtained from the South Central – Oxford B NHS Research Ethics Committee (19/SC/0212) and was approved by the HRA and Health and Care Research Wales, prior to the recruitment of participants commencing at any NHS site. We will disseminate our findings to academics and researchers through high

For peer review only - http://bmjopen.bmj.com/site/about/guidelines.xhtml

impact open access publications and through presentations at relevant academic and clinical conferences. Results will be made available to all participants after the completion of the study.

DISCUSSION

There is little research evaluating the effectiveness and cost-effectiveness of interventions in the treatment of self-harm in adolescents. The development of mHealth apps to support mental health interventions for adolescents is novel and, whilst technology offers many potential benefits, there is a lack of evidence regarding their efficacy, safety and acceptability [24].

A concern about the use of digital technologies to improve mental health relates to levels of user engagement, uptake and adherence [47]. A process of co-design involving users and research evidence is more likely to produce an intervention that is evidence-based, engaging, acceptable and feasible to deliver [48]. BlueIce was developed and co-designed with young people with a lived experience of self-harm where young people advised on all aspects of app development including design, layout, flow, and content [27]. Workshops with clinicians ensured that the mood-lifting techniques reflected both the recommended interventions and clinical practice. The results from our initial open study are encouraging [27]. Whilst they suggest that BlueIce is engaging and appealing to young people a more robust analysis is required to substantiate these findings.

Finally, this project will evaluate the use of BlueIce as a prescribed app, used in conjunction with a specialist mental health intervention. Future research will be required to evaluate the use of BlueIce as a standalone, freely accessed self-help app, or the mechanisms underpinning any effects.

For peer review only

TRIAL STATUS

Recruitment began in January 2020 and, pending COVID, will end around February 2022.

ACKNOWLEDGEMENTS

This project is funded by the National Institute for Health Research (NIHR) under its Research for Patient Benefit (RfPB) Programme (Grant Reference Number NIHR/PB-PG-1217-20004). The views expressed are those of the author(s) and not necessarily those of the NIHR or the Department of Health and Social Care.

FUNDING

This study was funded by the National Institute for Health Research, Research for Patient Benefit programme (PB-PG-1217-20004).

AUTHOR'S CONTRIBUTION

PS, GT, AML and SR designed and obtained funding for the study. PS, IG and JT drafted the study protocol. All authors contributed to and approved the final version.

ETHICS APPROVAL

The project has been reviewed and approved by the South Central – Oxford B NHS Research Ethics Committee (19/SC/0212) and approved by the HRA and Health and Care Research Wales.

COMPETING INTERESTS

None.

For peer review only

For peer review only - http://bmjopen.bmj.com/site/about/guidelines.xhtml

REFERENCES

1. Klonsky, E. D. The functions of deliberate self-injury: a review of the evidence. *Clinical Psychology Review*. 2007;27(2):226-239.
2. Samaritans, 2019. Samaritans Scotland: Reducing Self-Harm Key To Suicide Prevention [online]. Available from: https://www.samaritans.org/news/samaritans-scotland-reducing-self-harm-key-suicide-prevention/ [last accessed 14 January 2021].
3. Swannell S V, Martin G E, Page A, et al. Prevalence of Nonsuicidal Self-Injury in Nonclinical Samples: Systematic Review, Meta-Analysis and Meta-Regression. *The Journal of Child Psychology and Psychiatry*. 2014;44(3).
4. Plener P L, Schumacher T S, Munz L M, et al. The longitudinal course of non-suicidal self-injury and deliberate self-harm: a systematic review of the literatures. *Borderline Personality Disorder and Emotion Dysregulation*. 2015;30(2):2. doi: 10.1186/s40479-014-0024-3.
5. Klonsky E D. Nonsuicidal Self-Injury: What We Know, and What We Need to Know. *Canadian journal of psychiatry*. 2014;59(11):565-568.
6. Geulayov G, Casey D, McDonald K C, et al. Incidence of suicide, hospital-presenting non-fatal self-harm, and community-occurring non-fatal self-harm in adolescents in England (the iceberg model of self-harm): a retrospective study. *Lancet Psychiatry*. 2018;5(2):167-174.
7. Hawton K, Saunders K E, O'Connor R C. Self-harm and suicide in adolescents. *Lancet*. 2012;379(9834):2373-2382.[doi: 10.1016/S0140-6736(12)60322-5] [Medline: 22726518]
8. World Health Organization, 2019. Suicide [online]. Available from: https://www.who.int/news-room/fact-sheets/detail/suicide [last accessed 14 January 2021].
9. Hawton K, Zahl D, & Weatherall R. Suicide following deliberate self-harm: Long-term follow-up of patients who presented to a general hospital. *The British Journal of Psychiatry*. 2003:6:537–542. 10.1192/bjp.182.6.537
10. Cipriano A, Cella S, Cotrufo, P. Nonsuicidal Self-injury: A Systematic Review. *Frontiers in Psychology*. 2017;8(8):1946. doi: 10.3389/fpsyg.2017.01946.
11. Huen J M Y, Ip B Y T, Ho S M Y, et al. Hope and Hopelessness: The Role of Hope in Buffering the Impact of Hopelessness on Suicidal Ideation. *PLoS ONE*. 2015;10(6). doi: 10.1371/journal.pone.0130073
12. Stead V E, Boylan K, Schmidt, L. A. Longitudinal associations between non-suicidal self-injury and borderline personality disorder in adolescents: a literature review. *Borderline Personality Disorder and Emotion Dysregulation*. 2019;13(6):3. doi: 10.1186/s40479-019-0100-9
13. Hawton K, Witt K G, Salisbury T. L. T. et al. Interventions for self-harm in children and adolescents. *Cochrane Systematic Review*. 2015. doi: 10.1186/s40479-014-0024-3.
14. Lyengar U, Snowden N, Asamow J R, et al. A Further Look at Therapeutic Interventions for Suicide Attempts and Self-Harm in Adolescents: An Updated Systematic Review of Randomized Controlled Trials. *Frontiers in Psychiatry*. 2018;23(9):583. doi: 10.3389/fpsyt.2018.00583.

For peer review only - http://bmjopen.bmj.com/site/about/guidelines.xhtml

15. Cottrell D J, Wright-Hughes A, Collinson, M, et al. Effectiveness of systemic family therapy versus treatment as usual for young people after self-harm: a pragmatic, phase 3, multicentre, randomised controlled trial. *The Lancet*. 2018;5(3):203-216.
16. Linehan M M. Cognitive-Behavioural Treatment of Borderline Personality Disorder. New York:Guilford Publications. 2018.
17. Mehlum L, Ramberg M, Tormoen A J, et al. Dialectical Behaviour Therapy Compared with Enhanced Usual Care for Adolescents with Repeated Suicidal and Self-Harming Behaviour: Outcomes Over a One-Year Follow-Up. *Journal of the American Academy of Child & Adolescent Psychiatry*. 2016:55(4):295-300.
18. Mehlum L, Ramleth R, Tormoen A J, et al. Long term effectiveness of dialectical behavior therapy versus enhanced usual care for adolescents with self-harming and suicidal behavior. *Journal of Child Psychology and Psychiatry*. 2019;60(10). https://doi.org/10.1111/jcpp.13077
19. McCauley E, Berk M S, Asarnow J. R, et al. Efficacy of Dialectical Behaviour Therapy for Adolescents at High Risk for Suicide: A Randomized Clinical Trial. *JAMA Psychiatry*. 2018;75(8):777-785.
20. Rossouw T I, Fonagy P. Mentalization-based treatment for self-harm in adolescents: a randomized controlled trial. *Journal of the American Academy of Child & Adolescent Psychiatry.* 2012;51(12):1304-1313.
21. NHS England, 2017. Next steps on the NHS five year forward view [online]. Available from: https://www.england.nhs.uk/publication/next-steps-on-the-nhs-five-year-forward-view/. [last accessed 25 March 2020].
22. Grist R, Porter J, Stallard P. Mental Health Mobile Apps for Preadolescents and Adolescents: A Systematic Review. *Journal of Medical Internet Research*. 2017;19(5). doi:10.2196/jmir.7332.
23. Office of Communications, 2020. Children and parents: Media use and attitudes report 2019 [online]. Available from: https://www.ofcom.org.uk/__data/assets/pdf_file/0023/190616/children-media-use-attitudes-2019-report.pdf. [last accessed 16 April 2020].
24. Melia R, Francis K, Hickey E, et al. Mobile Health Technology Interventions for Suicide Prevention: Systematic Review. *JMIR Publications*. 2020;8(1).
25. Rasing S P A, Stikkelbroek Y A J, Bodden D H M. Is Digital Treatment the Holy Grail? Literature Review on Computerized and Blended Treatment for Depressive Disorders in Youth. *International Journal of Environmental Research and Public Health*. 2020;17(153).
26. Stallard P, Porter J, Grist R. Safety, acceptability, and use of a smartphone app, BlueIce, for young people who self-harm: protocol for an open phase I trial. *JMIR research protocols.* 2016;5(4), e217.
27. Stallard P, Porter J, Grist R. A smartphone app (BlueIce) for young people who self-harm: open phase 1 pre-post trial. *JMIR mHealth and uHealth*. 2018;6(1), e32.
28. NHS Digital Developer, 2019. Developer.nhs.uk. Digital Assessment Questions – Beta [online]. Available from: https://developer.nhs.uk/digital-tools/daq/. [last accessed 13 April 2020]
29. Vrouva I, Fonagy P, Fearon P, et al. The Risk-Taking and Self-Harm Inventory for Adolescents (RTSHIA): Development and Psychometric Evaluation. *Psychological Assessment*. 2010;22(4):852-865. doi:10.1037/a0020583.

For peer review only - http://bmjopen.bmj.com/site/about/guidelines.xhtml

30. Kidger J, Heron J, Lewis G, et al. Adolescent self-harm and suicidal thoughts in the ALSPAC cohort: a self-report survey in England. *BMC Psychiatry*. 2012;12(69). https://doi.org/10.1186/1471-244X-12-69
31. Costello E J, Angold A. Scales to Assess Child and Adolescent Depression: Checklists, Screens, and Nets. *Journal of the American Academy of Child & Adolescent Psychiatry*. 1988;27(6): 726-737.
32. Beck A T, Weissman A, Lester D, et al. The measurement of pessimism: the hopelessness scale. *Journal of consulting and clinical psychology*. 1974;42(6):861-865.
33. Kazdin A E, French N H, Unis A S, et al. Hopelessness, depression, and suicidal intent among psychiatrically disturbed inpatient children. *Journal of Consulting and Clinical Psychology*. 1983;51(4):504-510. https://doi.org/10.1037/0022-006X.51.4.504
34. Chorpita B F, Yim L, Moffitt C, et al. Assessment of symptoms of DSM-IV anxiety and depression in children: a revised child anxiety and depression Scale. *Behavior Research and Therapy.* 2000;38:835–855. doi: 10.1016/S0005-7967(99)00130-8.
35. Goodman R. The Strengths and Difficulties Questionnaire: A Research Note. *Journal of Child Psychology and Psychiatry*. 1997;38(5):581-586.
36. Espie C A, Kyle S D, Hames P, et al. The Sleep Condition Indicator: a clinical screening tool to evaluate insomnia disorder. *BMJ open*. 2014;4(3). e004183.
37. Grist R, Porter J, Stallard P. Acceptability, Use, and Safety of a Mobile Phone App (BlueIce) for Young People Who Self-Harm: Qualitative Study of Service Users' Experience. *JMIR mental health*. 2018;5(1).
38. Stevens K J. Assessing the performance of a new generic measure of health related quality of life for children and refining it for use in health state valuation. *Applied Health Economics and Health Policy.* 2011; 9(3): 157-169.
39. Ratcliffe J, Stevens K, Flynn T,et al. An assessment of the construct validity of the CHU9D in the Australian adolescent general population. *Quality of Life Research.* 2012;21(4):717-25.
40. Furber G, Segal L. The validity of the Child Health Utility instrument (CHU9D) as a routine outcome measure for use in child and adolescent mental health services. *Health and quality of life outcomes*. 2015;13(1):1.
41. Drummond M F, Sculpher M J, Claxton K, et al. Methods for the economic evaluation of health care programmes. Oxford: Oxford university press. 2015.
42. Husereau D, Drummond M, Petrou S, et al. Consolidated health economic evaluation reporting standards (CHEERS) statement. *BMJ*. 2013;346:f1049.
43. Briggs A, Fenn P. Confidence intervals or surfaces? Uncertainty on the cost-effectiveness plane. *Health economics*.1998;7(8):723-740.
44. Fenwick E, Byford S. A guide to cost-effectiveness acceptability curves. *BJ Psych*. 2005;187(2):106-108.
45. Faria R, Gomes M, Epstein D, et al. A guide to handling missing data in cost-effectiveness analysis conducted within randomised controlled trials. *Pharmacoeconomics*. 2014;32(12):1157-1170.
46. Schulz K F, Altman D G, Moher D, for the CONSORT Group. CONSORT 2010 Statement: updated guidelines for reporting parallel group randomised trials. *Lancet*. 2010;375(9721):1136.

For peer review only - http://bmjopen.bmj.com/site/about/guidelines.xhtml

47. Bevan Jones R, Stallard P, Agha SS, Rice S, Werner-Seidler A, Stasiak K, Kahn J, Simpson SA, Alvarez-Jimenez M, Rice F, Evans R. Practitioner review: Co-design of digital mental health technologies with children and young people. Journal of Child Psychology and Psychiatry. 2020 Aug;61(8):928-40.
48. Thabrew H, Fleming T, Hetrick S, Merry S. Co-design of eHealth interventions with children and young people. Frontiers in psychiatry. 2018 Oct 18;9:481.

For peer review only

For peer review only - http://bmjopen.bmj.com/site/about/guidelines.xhtml

BMJ Open

Beating Adolescent Self harm (BASH). A randomised controlled trial comparing usual care versus usual care plus a smartphone self-harm prevention app (BlueIce) in young adolescents aged 12-17 who self-harm: study protocol.

Journal:	*BMJ Open*
Manuscript ID	bmjopen-2021-049859.R1
Article Type:	Protocol
Date Submitted by the Author:	23-Jun-2021

Complete List of Authors:	Greenhalgh, Isobel; NHS, Child and Adolescent Mental Health Services<br>Tingley, Jessica; NHS, Child and Adolescent Mental Health Services<br>Taylor, Gordon; University of Exeter, College of Medicine and Health<br>Medina-Lara, Antonieta; University of Exeter Medical School, Health Economics Group<br>Rhodes, Shelley; University of Exeter, Medical School<br>Stallard , P; NHS, Child and Adolescent Mental Helth Services ; University of Bath, Health

**Primary Subject Heading**:	Mental health
Secondary Subject Heading:	Mental health, Paediatrics, Health services research
Keywords:	MENTAL HEALTH, Telemedicine < BIOTECHNOLOGY & BIOINFORMATICS, Suicide & self-harm < PSYCHIATRY, Child & adolescent psychiatry < PSYCHIATRY

SCHOLARONE™
Manuscripts

I, the Submitting Author has the right to grant and does grant on behalf of all authors of the Work (as defined in the below author licence), an exclusive licence and/or a non-exclusive licence for contributions from authors who are: i) UK Crown employees; ii) where BMJ has agreed a CC-BY licence shall apply, and/or iii) in accordance with the terms applicable for US Federal Government officers or employees acting as part of their official duties; on a worldwide, perpetual, irrevocable, royalty-free basis to BMJ Publishing Group Ltd ("BMJ") its licensees and where the relevant Journal is co-owned by BMJ to the co-owners of the Journal, to publish the Work in this journal and any other BMJ products and to exploit all rights, as set out in our licence.

The Submitting Author accepts and understands that any supply made under these terms is made by BMJ to the Submitting Author unless you are acting as an employee on behalf of your employer or a postgraduate student of an affiliated institution which is paying any applicable article publishing charge ("APC") for Open Access articles. Where the Submitting Author wishes to make the Work available on an Open Access basis (and intends to pay the relevant APC), the terms of reuse of such Open Access shall be governed by a Creative Commons licence – details of these licences and which Creative Commons licence will apply to this Work are set out in our licence referred to above.

Other than as permitted in any relevant BMJ Author's Self Archiving Policies, I confirm this Work has not been accepted for publication elsewhere, is not being considered for publication elsewhere and does not duplicate material already published. I confirm all authors consent to publication of this Work and authorise the granting of this licence.

For peer review only

Beating Adolescent Self harm (BASH). A randomised controlled trial comparing usual care versus usual care plus a smartphone self-harm prevention app (BlueIce) in young adolescents aged 12-17 who self-harm: study protocol.

Greenhalgh I[1], Tingley J[1], Taylor G[2], Medina-Lara A[3], Rhodes S[2], & Stallard P[1].

Author Information

1. Child and Adolescent Mental Health Service (CAMHS), NHS, Keynsham, UK
2. College of Medicine and Health, University of Exeter, Exeter, UK
3. Clinical Trials Unit, University of Exeter, Exeter, UK
4. Department for Health, University of Bath, Bath, UK

Full name, postal address, and email of the corresponding author:

Paul Stallard, 6.10 Wessex House, Department of Health, University of Bath, BA2 7AY, UK.
P.stallard@bath.ac.uk

Word count: 5141.

For peer review only

ABSTRACT

Introduction

A mobile app, BlueIce, was co-designed with young people with a history of self-harm to provide them with more accessible and available evidence-based support at times of distress. A preliminary evaluation found that BlueIce was acceptable, safe, and used by young people, and helped to reduce self-harm. The present study is designed to assess the effectiveness and cost-effectiveness of adding BlueIce to usual child and adolescent mental health care (CAMHS).

Methods and analysis

This study is a single-blind, randomised controlled trial comparing usual CAMHS care with usual care plus BlueIce. A total of 138 adolescents aged 12-17 with current or a history of self-harm will be recruited through the Oxford Health NHS Foundation Trust via their CAMHS clinician. The primary outcome is self-harm at 12 weeks assessed using the Risk Taking and Self-Harm Inventory for Adolescents (RTSHIA). Secondary outcomes include mood, anxiety, hopelessness, general behaviour, sleep and impact on everyday life at 12 weeks and 6 months. Health related quality of life and health care resource utilisation data will be collected at baseline, 12 weeks, and 6 months. Post-use interviews at 12 weeks will determine the acceptability, safety, and usability of BlueIce.

Ethics and dissemination

The study was approved by the NHS South Central – Oxford B NHS Research Ethics Committee (19/SC/0212) and by the HRA and Health and Care Research Wales. Findings will be disseminated in peer review open-access journals and at academic conferences.

Trial Registration number

ISRCTN10541045.

Strengths and limitations of this study

- This is the first randomised controlled trial to explore the effects of adding a self-help app (BlueIce) to usual care on the self-harm of adolescents receiving specialist mental health care.
- The study includes a detailed economic analysis to determine the cost-effectiveness of BlueIce
- The BlueIce app was co-designed with young people with a lived experience of self-harm.
- Participants will not be blinded to participant group as this is prohibited by the nature of the intervention.

For peer review only

Trial registration: ISRCTN registry, registration number ISRCTN 10541045. Registered on 22/07/2019

Keywords

BlueIce, Self-Harm, Children, Adolescents, Mobile App. CAMHS, Mental Health, (m)Health

BACKGROUND

Self-harm is defined as the deliberate act of causing damage to one's body, for example through self-poisoning or self-cutting [1,2]. It is very prevalent, with around 17.2% of adolescents and 13.4% of young adults being estimated to self-harm [3]. Rates of self-harm are more likely to reduce in young adults compared with younger adolescents [4]. Most self-harm occurs in secret with comparatively few episodes resulting in hospital presentations [5-7]. The most common reasons for self-harm include tension relief, escape from intolerable psychological pain, self-punishment and to show others how bad they are feeling (7). While suicide is less prevalent amongst younger adolescents, it is the third most common cause of death in young people [8]. Self-harm has consistently been cited a risk factor for later suicide attempts [9]. Studies have demonstrated that adolescent non-suicidal self-harm is a strong predictor of future suicide attempts in young adults [10]. Findings such as these highlight the importance of intervening with those who are self-harming in preventing future suicides [11]

Research has identified risk factors for self-harm, including: negative and stressful life events such as childhood maltreatment; self-harm or attempted suicide of a family member; drug and alcohol abuse; psychological factors such as feelings of hopelessness, perfectionist traits, emotional dysregulation, or diagnoses of psychological disorders; lower sociodemographic status; and being female [7,12-14]. Protective factors which may help reduce or prevent self-harm include better access to social support, higher levels of self-esteem and receiving support from parents, as well as being able to re-evaluate one's own thoughts and beliefs [4].

Evidence-based interventions for self-harm in children and adolescents are scarce. Few studies have evaluated cognitive behaviour therapy (CBT) or problem-solving [15,16]. Family therapy does not appear effective [17]. There is some support for dialectical behaviour therapy (DBT) [18-21], and for mentalization-based treatment (MBT) [22]. However, a Cochrane review concluded that there is not much evidence on which to draw conclusions on the effects of interventions for adolescent self-harm and recommended that therapeutic assessment, metallization, dialectical behaviour, and cognitive behaviour therapy warrant further evaluation [15]. Recent reviews have identified new studies evaluating DBT with adolescents and suggest that DBT does now meet criteria for a well-established treatment for self-harm [23]. With recent advances in technology the use of digital interventions to aid the delivery of mental health interventions has become more widespread. The NHS is encouraging the use of technology to improve access to, and the availability of, support and interventions, and to reduce demands on mental health services [24].

For peer review only

For peer review only - http://bmjopen.bmj.com/site/about/guidelines.xhtml

There are currently over 15,000 mobile (m)health apps available worldwide, with around 5,000 of these targeted at mental health [25]. Apps offer an accessible way of delivering and supporting mental health interventions for young people since over 80% of 12-15-year-olds own a smartphone [26]. Alongside improving accessibility to support, mHealth apps are available 24/7, provide a means for symptom monitoring between face-to-face meetings, may be a preferable method of accessing support for some young people, and provide a route around barriers such as stigma. mHealth apps may also help to lessen the demand on healthcare resources [25,27,28]. However, the evidence base surrounding healthcare apps is severely lacking, with research unable to keep pace with the speed of their development [27]. Therefore, the safety, efficacy, and acceptability of most apps is unknown.

In terms of self-harm and suicide prevention, mhealth appears very acceptable and appealing to young people and offers a way of providing immediate support at times of crisis. However, hardly any suicide or self-harm prevention apps have been developed with a recent systematic review identifying only four [29]. Similarly, systematic reviews evaluating the efficacy of technology and mhealth interventions in preventing suicide or self-harm in adolescents [30] and university students [31] have raised similar concerns. In addition, the availability of self-harm prevention apps is extremely limited with few being available for clinical use [31]. Whilst these reviews all highlight the potential of self-help apps, further research is required to determine their efficacy in self-harm and suicide prevention with clinical populations.

In response to the above, a self-help app, BlueIce, was co-developed with young people with a lived experience of self-harm [32]. It is designed to support self-management of distress and reduce self-harm behaviours. BlueIce has 24/7 accessibility and is a prescribed app to be used alongside face-to-face Child and Adolescent Mental Health Services (CAMHS) [33]. BlueIce is password protected and all data is stored locally on the phone [32]. It meets all minimum standards required for NHS accredited apps [34].

An initial open study to determine safety, acceptability and usability found that 73% (19/26) of BlueIce users reported a reduction or cessation of self-harm at the 12-week follow-up [27]. No participants reported an increase in self-harm during the study. Post-use reductions in symptoms of anxiety and depression were also reported with 88% (29/33) of users electing to keep the app at the end of the study [33].

The aims of this trial are to determine the effectiveness, cost- effectiveness and acceptability of adding BlueIce to usual face-to-face specialist mental health care in the reduction of self-harm in adolescents.

For peer review only - http://bmjopen.bmj.com/site/about/guidelines.xhtml

METHODS

Trial design

This is a two arm, single blind, randomised controlled trial (RCT) comparing the addition of the BlueIce self-help app to usual face-to-face specialist mental health care (UC+BI) with usual specialist mental health care (UC).

Setting and participants

Participants will be recruited through CAMHS provided by Oxford Health NHS Foundation Trust, covering Bath and North-East Somerset, Swindon, Wiltshire, Buckinghamshire, and Oxfordshire.

Young people will be eligible if they: (1) are receiving treatment from CAMHS at the time of referral; (2) have self-harmed at least twice in the last 12 months; and (3) are aged between 12 and 17 years.

Exclusion criteria are: (i) a diagnosis of psychosis; (ii) a significant learning disability which would interfere with the young person's ability to use the app; (iii) young people with active suicidal plans, or (iv) safeguarding concerns where the young person has suffered abuse within the last 6 months or is the subject of a safeguarding investigation.

Recruitment

Clinicians across Oxford Health NHS Foundation Trust will be invited to identify eligible young people who are open to CAMHS. Clinicians will discuss the study and provide interested young people, and if under 16 their parents/carers, with a project information sheet. If interested in participating, their details will be forwarded to the research team.

Consent

Researchers will meet with the young people, and if appropriate their parents/carers, to discuss the project. If the young person wants to take part, the researcher will then obtain consent. If under 16 years old, the young person will be asked to provide assent while their parent or legal guardian will be asked for consent. Those aged 16 years or older will be able to provide their own signed consent. During the COVID pandemic the consent and assessment process will be undertaken remotely (i.e., online or via telephone) to maintain the safety of participants and the research team.

Randomisation

Computer generated randomisation will be independently undertaken by Exeter Clinical Trials Unit. Participants will be randomised in a 1:1 ratio to either usual specialist mental health care (UC) or usual specialist mental health care plus the BlueIce self-help app (UC+BI). Participants will be randomised using REDCap software minimising for gender, age

For peer review only - http://bmjopen.bmj.com/site/about/guidelines.xhtml

(over or under 16), self-harm frequency in last 4 weeks (0-2 or ≥3), and severity of depression (Mood and Feelings Questionnaire above or below 27, the cut off for severe depression). Because of the nature of the intervention, participants will not be blind to their allocation. However, researchers involved in data collection will remain blind to allocation. Participant allocation will be undertaken by a member of the project team who is not involved in undertaking patient assessments.

Interventions

Usual Care (UC): Young people will receive individual mental health interventions from specialist CAMHS clinicians. This will be either face-to-face, or, due to COVID, a remotely delivered telephone or video intervention. The nature, content, and duration of this will be captured by the health care resource questionnaire.

Usual care plus BlueIce (UC+BI): In addition to usual care, young people will also receive access to the self-help BlueIce app.

BlueIce is an application for android and apple smartphones. It contains a mood diary, personalised toolbox of mood lifting strategies that are available to the young person 24/7 and automatic routing to emergency contact numbers.

(i). *Mood diary*: Upon entering BlueIce, young people rate their mood. For each mood rating the young person has the option of adding a note to record any particular reason why they might be feeling as they do. Their rating and notes are saved in a calendar which the young person and therapist can review to look for changes and patterns over time. (ii). *Mood lifting*: If the young person rates their mood as low, they will automatically be routed to the mood lifting section. Alternatively, if at any time the young person would like to access this section they can do so directly from the main menu. This section contains a menu of mood lifting and distress tolerance activities, personalised according to the interests of the young person. The activities are designed to counter the common reasons why young people self-harm (to punish themselves, emotional relief, feeling hopeless) and draws on common methods used in cognitive behaviour therapy (CBT) and dialectical behaviour therapy (DBT). The mood lifting section includes 8 activities: 1). Photo library: the young person can upload and save photographs, inspirational quotes and pictures that are associated with happy memories or which might make them feel good. These can be reviewed when low to help the young person remember the positive things in their life: 2). Music library: a music player is included where the young person can upload and store music they enjoy, and which has a positive effect on how they feel. This playlist can be readily accessed when the young person is low as a way of improving their mood: 3). Physical activities: the young person can identify physical activities they enjoy such as sporting activities (e.g., going for a run or riding a bike) or other aerobic activities such as walking the dog. The young person can access their personalised list when low and be reminded about what they can do to get active to improve their mood: 4). Mood changing activities: BlueIce includes a section of activities that make the young person feel good. These could be things like making a cake, watching an episode of a favorite TV series, reading a book, playing with a pet. These provide the young person with a prompt list of activities they can use to change their mood when feeling down: 5). Relaxation and mindfulness exercises: audio-recorded instructions for a 10-minute

For peer review only - http://bmjopen.bmj.com/site/about/guidelines.xhtml

mindfulness session, calming visualisation and a quick controlled breathing exercise (4-7-8 breathing) are included. These can be used to help the young person manage any unpleasant emotions or distressing thoughts: 6). Identification of negative thoughts: this section includes a thought diary where the young person can record any troubling thoughts that are racing through their head. These can be directly typed into BlueIce where they are saved and can be reviewed at a later date. This allows identification of any themes that could be addressed during face-to-face work with their clinician: 7). Ride it out: this section draws on ideas from DBT and helps the young person to tolerate their distress. This includes instructions for an ice dive, a sensory toolbox and a “pros and cons” balance sheet for self-harming: 8). Call a friend: the final section contains the phone numbers of 3-5 people who the young person could contact if they were feeling low and in danger of self-harming. These would be people who make them feel happy and those they could talk with about how they are feeling. This section prompts the young person to reach out to others. (iii) *Emergency contacts*. After accessing the mood lifter, the young person is asked to re-rate their mood. If they are still low and feeling that they might harm themselves, they will be routed through a series of questions to three emergency contact numbers. The young person can select one of these options to automatically call/text emergency support.

Patient and public involvement

BlueIce was co-designed and produced with young people with a lived experience of self-harm. They were involved in exploring the concept (would an app be helpful?), what an app should look like (examples of apps liked and used), the design (font, colours, flow) and content (evidence based and ideas young people found helpful).

In this study, two young people will be recruited to join our Study Steering Committee (SSC). We plan participant workshops to develop study resources, to advise on recruitment and retention issues, to discuss study findings, identify key messages, prepare understandable research summaries in different formats, and identify issues/implications for future research. We intend to involve young people in events disseminating the findings of the study.

Assessment schedule

Data will be collected at: (i) baseline; (ii) post-intervention (12 weeks); and, (iii) follow-up (6 months after randomisation). Data will be collected by Research Assistants, blind to treatment allocation. Participants will be given a £20 voucher after completing the final assessment.

Baseline: Standardised self-report measures of self-harm, depression, anxiety, hopelessness, general behaviour, impact on everyday life, sleep and health-related quality of life and resource use health care questionnaire. These will be complemented with case-note review: to detail resource use i.e., accident and emergency department attendances, out of hours contacts, primary and secondary care attendances following incidents of self-harm in the preceding 6 months.

Post-intervention (12 weeks): Standardised self-report measures of self-harm, depression, anxiety, hopelessness, general behaviour, impact on everyday life, sleep and health-related

For peer review only - http://bmjopen.bmj.com/site/about/guidelines.xhtml

quality of life and the resource use health care questionnaire will be repeated (baseline -12 weeks). Those in UC + BI will complete a semi-structured interview detailing their use, experience of, and satisfaction with BlueIce.

Follow-up (6 months after randomisation): Standardised self-report measures of self-harm, depression, anxiety, hopelessness, general behaviour, impact on everyday life, sleep and health-related quality of life. Case note review will be repeated (12 weeks to 6 months) and the type and total hours of direct and indirect CAMHS intervention provided from randomisation to 6 months detailed.

Outcome measures

Primary Outcome: Our assessment of self-harm will consist of three parts: A). a brief interview; B). completion of the Risk Taking and Self-Harm Inventory for Adolescents (RTSHIA) [35] and C). the provision of support and advice.

Part A: Interview. Young people will be asked “have you ever hurt yourself on purpose in any way (e.g., by taking an overdose of pills or by cutting yourself) over the past 3 months?” which was taken from the Avon Longitudinal Study of Parents and Children (ALSPAC) (http://www.alspac.bris.ac.uk) [36]. Young people are asked to consider the last 6 months at baseline, and the last 3 months at follow-ups. Those who answer yes will be asked further questions about frequency, method, reason for self-harming, whether they sought medical help and suicidal intent.

Part B: RTSHIA. Our primary outcome is self-reported self-harm assessed by the self-harm inventory of the RTSHIA. The RTSHIA was developed in the UK for use with adolescents (aged 11-19 years) [35]. The self-harm inventory consists of 18 items and assesses the presence and frequency of a range of intentional self-injuries (e.g., cutting, burning, self-hitting, self-poisoning). The frequency of each item is rated on a 4-point scale (never, once, more than once, many times) over a defined period. At baseline, the young person is asked to consider the 6 months before their initial assessment. For the 12 week and 6-month assessment the young person considers the 3-month period since their last assessment. Each item is then scored (0,1,2,3) and totalled to provide a current self-harm score. The RTSHIA has good reliability and validity [22,35]. We will analyse total scores and use this information to categorise changes in self-harm from baseline to 12 weeks and 6 months as reduced/stopped vs same/increased.

Part C: Support and Advice. At the end of the assessment young people will be given a list of contacts they can call if they are feeling worried about themselves. These include NHS 111, Childline and the Samaritans.

Secondary outcomes: The Mood and Feelings Questionnaire (MFQ) [37], is a self-report questionnaire for depression recommended by NICE consisting of 33 items rated as either “true” (scores 2), “sometimes true” (scores 1) or not true (scores 0). The MFQ has high criterion validity and correlates well with other measures of depression [37]. A total score of 27 and above is associated with major depression, 20 with mild depression and 16 with no mood disorder. The Hopelessness Scale for children, adapted from Beck’s Hopelessness Scale [38,39], consists of 17 true–false items measuring hopelessness and negative

For peer review only - http://bmjopen.bmj.com/site/about/guidelines.xhtml

expectations for the future. Items endorsed as "true" are summed, with higher scores indicating greater hopelessness. The Hopelessness Scale for children has been widely used within adolescent samples and has consistently demonstrated strong psychometric properties [38,39]. The Revised Child Anxiety and Depression Scale (RCADS) [40] is a 47-item questionnaire with items corresponding to DSM-IV criteria for anxiety in the areas of social phobia, separation anxiety, obsessive compulsive disorder, panic disorder, generalised anxiety disorder and for major depressive disorder. Each item is rated on a 4-point Likert scale of frequency (never 0; sometimes 1; often 2; always 3) which are summed to produce sub-scale and total anxiety scores. The Strengths and Difficulties Questionnaire (SDQ) [41] is a widely used behavioural screening questionnaire consisting of 25 items assessing emotional symptoms, conduct problems, hyperactivity and/or inattention, peer relationship problems, and pro-social behaviour. Each item is rated as not true (0), somewhat true (1) or certainly true (2). A total difficulties score is calculated by summing scores from all subscales except the pro-social. In addition, an impact supplement assesses the degree of distress created by the child's difficulties and the degree to which they interfere with home life, friendships, classroom leaning and leisure activities. The five items are summed to produce a total 'impact on everyday life' score which ranges from 0-10. The Sleep Condition Indictor (SCI) [42] is an eight item self-report measure assessing sleep and impact on daytime functioning over the past month on a 5-point scale. Item scores are summed to produce a total score ranging from 0-32. The SCI is an internally consistent ($\alpha = .86$) measure with a clinical cut-off <17 correctly identifying 89% of those with probable DSM-5 insomnia disorder [42].

Qualitative evaluation: We will use the semi-structured interview developed in our initial study [43] to assess participant's experience of BlueIce including use, ratings of satisfaction, helpfulness, ease of use and whether they would recommend it to a friend. In addition, we will assess the degree to which BlueIce was used and which parts of the app were used most often. This will only be completed by UC+BI at 12 weeks.

Economic Analysis: A cost-effectiveness analysis will be undertaken alongside the RCT to estimate the incremental cost-effectiveness of usual care + Blue Ice (UC+BI) compared to usual care (UC) i.e., incremental cost per unit of health outcome (primary outcome) and Quality Adjusted Life Years (QALYs) using the NHS and social care perspective. In order to estimate QALYs we will use the Child Health Utility 9D (CHU-9D). This preference-based generic HRQoL measure is designed specifically for use in the economic evaluation of health care interventions in young people [44]. The CHU-9D contains nine dimensions (worried, sad, pain, tired, annoyed, schoolwork/homework, sleep, daily routine and activities), each with five levels of functioning rated for "today". The CHU-9D has been validated for self-completion by young people (aged 7–17 years) [45] and with child and adolescent mental health services [46]. Resource use and cost data will be collected from the participants recruited in the RCT using a resource use questionnaire. Data on self-reported resource use will be compared with the self-harm assessment data that are recorded in clinical case notes (CareNotes). Clinical records will be reviewed for accident and emergency attendances, out of hours contacts, or primary and secondary care attendances following self-harm over two periods (6 months to baseline; baseline to 6 months). We will also quantify the number of face-to-face appointments and total number of hours of CAMHS input provided from baseline to 6 months. Incremental costs will be

For peer review only - http://bmjopen.bmj.com/site/about/guidelines.xhtml

combined with data on effectiveness/health outcomes. National unit costs will be obtained from available sources including PSSRU (https://www.pssru.ac.uk/) and from the National NHS cost collection (https://www.england.nhs.uk/national-cost-collection/). Analyses will follow good practice for conducting economic evaluations in health technology assessment [47] and findings will be reported using the CHEERS guidelines [48]. Results will include disaggregated data, as well as synthesis of cost and outcome data, and will include presentation of cost-effectiveness plane [49], cost-effectiveness acceptability curves [50], and detailed consideration of the broader impacts of the results reported. Robustness will be assessed through sensitivity analyses. Multiple imputation will be used to “fill-in” missing cost and outcome data, making the assumption that the data are missing at random [51]. If the young person is under 16, we will also ask their parent or guardian to complete both these questionnaires.

Sample size

A 3-point difference on our primary outcome (RTSHIA) between treatment groups represents a clinically important difference [35]. However, we propose to adopt a more conservative approach and will power the study to detect a moderate effect representing a 2-point difference. With a SD of 3.6, 90% power, alpha set at 0.05, we will require 69 participants per group.

Planned analysis

Statistical analysis

Our primary analysis will be at the end of the 12 weeks follow up of the last recruited participant. A Statistical Analysis Plan (SAP) will be developed by the trial statistician in consultation with the project management group, and agreed with the SSC before database lock. We will follow the CONSORT extension for reporting randomised controlled trials and will follow recommended guidelines for analysis of our data [52]. Our primary analysis at 12-weeks will be analysed on an intention to treat principle. Although we are not expecting a significant amount of missing data at 12 weeks, the impact of missing data will be assessed by comparing baseline covariates for missing and non-missing cases. In the event that there is evidence of bias being introduced into the analysis then further consideration, including but not limited to multiple imputation, will be given regarding how to address this.

Descriptive statistics will summarise baseline characteristics for each arm and patterns of missing follow-up data will be explored. We will also undertake a per protocol analysis of our primary outcome, total scores on the RTSHIA. Regression analysis adjusting for baseline minimisation variables of age, gender, mood and self-harm frequency will be undertaken. We will conduct sensitivity analyses in which we adjust for prognostic variables for which there is a baseline imbalance between intervention arms. Further sensitivity analyses will use multiple imputation to deal with missing data.

Similar regression analyses will be conducted for secondary outcomes (linear regression for numerical outcomes and logistic regression for binary outcomes). All secondary outcome measures will be compared between the groups and will include summary statistics and confidence intervals for measures of effect size.

For peer review only - http://bmjopen.bmj.com/site/about/guidelines.xhtml

Analysis of the 6-month data will be included in a repeated measures analysis to investigate the maintenance of any effect seen at 12 weeks. Analysis of the 6-month follow-up data will be undertaken using a repeated measures analysis of variance with both the 12 weeks and 6-month data being included and adjusted for the baseline. The analysis will also be adjusted for the baseline minimisation variables: age, gender, mood and frequency of self-harm as proposed for the primary analysis at 12 weeks.

Trial Management

An independent Study Steering Group (SSG) will be established to monitor progress, advise the investigators in general scientific and management issues and ensure that there are no major deviations from the study protocol. The SSG will include an independent chair, and at least 2 other independent members with research experience with young people with mental health problems and/or self-harm. The SSG will also include two young people from the Oxford Health participation group. The SSG will meet at least once per year. The lead applicant will inform the SSG Chair who may call additional meetings when there are matters arising from the conduct or management of the trial that might require their advice

A Data Monitoring Committee (DMC) was not convened as there were no panned interim analysis. Adverse events were reviewed by the SSG.

Adverse event reporting and harms

For the purposes of this trial, adverse events are defined as increases in extent of self-harm or suicidal ideation regardless of whether they are casually related to the trial procedures. Serious adverse events are those requiring hospital admission. All adverse and serious adverse events will be reported to the project leader and will be reviewed by the Study Steering Committee

Referring clinicians will be requested to inform study researchers should a young person experience an adverse event during their participation in the study. At both follow-up visits, the young person (and parent if under 16) will be asked if any adverse events have occurred and if they think the study is having any negative effects on their mental health and self-harm frequency.

Ethics and dissemination

Favorable ethical opinion for the research was obtained from the South Central – Oxford B NHS Research Ethics Committee (19/SC/0212) and was approved by the HRA and Health and Care Research Wales, prior to the recruitment of participants commencing at any NHS site. We will disseminate our findings to academics and researchers through high impact open access publications and through presentations at relevant academic and clinical conferences. Results will be made available to all participants after the completion of the study.

For peer review only - http://bmjopen.bmj.com/site/about/guidelines.xhtml

DISCUSSION

There is little research evaluating the effectiveness and cost-effectiveness of interventions in the treatment of self-harm in adolescents. Intervening with those who self-harm is important since self-harm during adolescence is a significant risk factor for future suicide attempts and completed episodes (10,11).The development of mHealth apps to support mental health interventions for adolescents offers a novel and accessible way of providing support at times of crisis. However, whilst technology offers many potential benefits, few apps to prevent self-arm have been developed and there is a lack of evidence regarding their efficacy, safety, and acceptability [25].

Strengths and limitations

This is the first adequately powered randomised controlled trial of a digital self-help app co-designed with, and used by, young people aged 12-17 years in receipt of specialist mental health services. This study will add to the evidence-base and will document the effectiveness and cost-effectiveness of adding a self-help app to usual care. If benefits are identified, BlueIce app can be widely made available to young people attending specialist CAMHS to provide help at times of distress.

As it is not feasible to blind participants to allocation, the research team are aware of the need to maintain equipoise and to present the two interventions to referring clinicians and participants in a balanced way. Researchers will remain blind to treatment arm and, if this is inadvertently broken, subsequent assessments will be conducted by another member of the research team. Similarly, following guidance from our participation group, those allocated to usual care will be provided with BlueIce at their final, six-month assessment.

We have minimised the possibility of contamination between the trial arms. BlueIce is a prescribed app and is not freely available to download and use. Those allocated to UC+BI will be sent a single use download code. Once activated, BlueIce will automatically be installed on the participant's smartphone and the access code will no longer work. Participants are therefore unable to share/pass the app/access code to others.

Finally, this project will evaluate the use of BlueIce as a prescribed app, used in conjunction with a specialist mental health intervention. Future research will be required to evaluate the use of BlueIce as a standalone, freely accessed self-help app, or the mechanisms underpinning any effects.

For peer review only - http://bmjopen.bmj.com/site/about/guidelines.xhtml

TRIAL STATUS

Recruitment began in January 2020 and, pending COVID, will end around June 2022.

TRIAL SPONSOR

Oxford Health NHS Foundation Trust. The sponsor will have no role in interpreting data, writing reports or decisions to publish findings.

ACKNOWLEDGEMENTS

This project is funded by the National Institute for Health Research (NIHR) under its Research for Patient Benefit (RfPB) Programme (Grant Reference Number NIHR/PB-PG-1217-20004). The views expressed are those of the author(s) and not necessarily those of the NIHR or the Department of Health and Social Care.

The authors would like to thank the Study Steering Group, Dr Patrick Smith, Professor Kapil Sayal. Dr Becky Mars and Mr Thom Walker for their advice, guidance, and support.

AUTHOR'S CONTRIBUTION

PS, GT, AML and SR designed and obtained funding for the study. PS, IG and JT drafted the study protocol. All authors contributed to and approved the final version.

FUNDING

This study was funded by the National Institute for Health Research, Research for Patient Benefit programme (PB-PG-1217-20004). The funder will have no role in interpreting data, writing reports or decisions to publish findings

ETHICS APPROVAL

The project has been reviewed and approved by the South Central – Oxford B NHS Research Ethics Committee (19/SC/0212) and approved by the HRA and Health and Care Research Wales.

COMPETING INTERESTS

BlueIce is the intellectual property of Paul Stallard, the creator of the app. He has no financial benefits from the app. The other authors have declared no interests.

For peer review only

For peer review only - http://bmjopen.bmj.com/site/about/guidelines.xhtml

REFERENCES

1. Klonsky, E. D. The functions of deliberate self-injury: a review of the evidence. Clinical Psychology Review. 2007;27(2):226-239.

2. Samaritans, 2019. Samaritans Scotland: Reducing Self-Harm Key To Suicide Prevention [online]. Available from: https://www.samaritans.org/news/samaritans-scotland-reducing-self-harm-key-suicide-prevention/ [last accessed 14 January 2021].

3. Swannell S V, Martin G E, Page A, et al. Prevalence of Nonsuicidal Self-Injury in Nonclinical Samples: Systematic Review, Meta-Analysis and Meta-Regression. The Journal of Child Psychology and Psychiatry. 2014;44(3).

4. Plener P L, Schumacher T S, Munz L M, et al. The longitudinal course of non-suicidal self-injury and deliberate self-harm: a systematic review of the literatures. Borderline Personality Disorder and Emotion Dysregulation. 2015;30(2):2. doi: 10.1186/s40479-014-0024-3.

5. Klonsky E D. Nonsuicidal Self-Injury: What We Know, and What We Need to Know. Canadian journal of psychiatry. 2014;59(11):565-568.

6. Geulayov G, Casey D, McDonald K C, et al. Incidence of suicide, hospital-presenting non-fatal self-harm, and community-occurring non-fatal self-harm in adolescents in England (the iceberg model of self-harm): a retrospective study. Lancet Psychiatry. 2018;5(2):167-174.

7. Hawton K, Saunders K E, O'Connor R C. Self-harm and suicide in adolescents. Lancet. 2012;379(9834):2373-2382.[doi: 10.1016/S0140-6736(12)60322-5] [Medline: 22726518]

8. World Health Organization, 2019. Suicide [online]. Available from: https://www.who.int/news-room/fact-sheets/detail/suicide [last accessed 14 January 2021].

9. Hawton K, Zahl D, & Weatherall R. Suicide following deliberate self-harm: Long-term follow-up of patients who presented to a general hospital. The British Journal of Psychiatry. 2003:6:537–542. 10.1192/bjp.182.6.537

10. Mars B, Heron J, Klonsky ED, Moran P, O'Connor RC, Tilling K, Wilkinson P, Gunnell D. Predictors of future suicide attempt among adolescents with suicidal thoughts or non-suicidal self-harm: a population-based birth cohort study. The Lancet Psychiatry. 2019 Apr 1;6(4):327-37.

11. Hawton K, Bergen H, Cooper J, Turnbull P, Waters K, Ness J, Kapur N. Suicide following self-harm: findings from the multicentre study of self-harm in England, 2000–2012. Journal of Affective Disorders. 2015 Apr 1;175:147-51.

12. Cipriano A, Cella S, Cotrufo, P. Nonsuicidal Self-injury: A Systematic Review. Frontiers in Psychology. 2017;8(8):1946. doi: 10.3389/fpsyg.2017.01946.

13. Huen J M Y, Ip B Y T, Ho S M Y, et al. Hope and Hopelessness: The Role of Hope in Buffering the Impact of Hopelessness on Suicidal Ideation. PLoS ONE. 2015;10(6). doi: 10.1371/journal.pone.0130073

14. Stead V E, Boylan K, Schmidt, L. A. Longitudinal associations between non-suicidal self-injury and borderline personality disorder in adolescents: a literature review. Borderline

For peer review only

Personality Disorder and Emotion Dysregulation. 2019;13(6):3. doi: 10.1186/s40479-019-0100-9

15. Hawton K, Witt K G, Salisbury T. L. T. et al. Interventions for self-harm in children and adolescents. Cochrane Systematic Review. 2015. doi: 10.1186/s40479-014-0024-3.

16. Lyengar U, Snowden N, Asamow J R, et al. A Further Look at Therapeutic Interventions for Suicide Attempts and Self-Harm in Adolescents: An Updated Systematic Review of Randomized Controlled Trials. Frontiers in Psychiatry. 2018;23(9):583. doi: 10.3389/fpsyt.2018.00583.

17. Cottrell D J, Wright-Hughes A, Collinson, M, et al. Effectiveness of systemic family therapy versus treatment as usual for young people after self-harm: a pragmatic, phase 3, multicentre, randomised controlled trial. The Lancet. 2018;5(3):203-216.

18. Linehan M. Cognitive-Behavioural Treatment of Borderline Personality Disorder. New York: Guilford Publications. 2018.

19. Mehlum L, Ramberg M, Tormoen A J, et al. Dialectical Behaviour Therapy Compared with Enhanced Usual Care for Adolescents with Repeated Suicidal and Self-Harming Behaviour: Outcomes Over a One-Year Follow-Up. Journal of the American Academy of Child & Adolescent Psychiatry. 2016:55(4):295-300.

20. Mehlum L, Ramleth R, Tormoen A J, et al. Long term effectiveness of dialectical behavior therapy versus enhanced usual care for adolescents with self-harming and suicidal behavior. Journal of Child Psychology and Psychiatry. 2019;60(10). https://doi.org/10.1111/jcpp.13077

21. McCauley E, Berk M S, Asarnow J. R, et al. Efficacy of Dialectical Behaviour Therapy for Adolescents at High Risk for Suicide: A Randomized Clinical Trial. JAMA Psychiatry. 2018;75(8):777-785.

22. Rossouw T I, Fonagy P. Mentalization-based treatment for self-harm in adolescents: a randomized controlled trial. Journal of the American Academy of Child & Adolescent Psychiatry. 2012;51(12):1304-1313.

23. Clarke S, Allerhand LA, Berk MS. Recent advances in understanding and managing self-harm in adolescents. F1000Research. 2019;8.

24. NHS England, 2017. Next steps on the NHS five year forward view [online]. Available from: https://www.england.nhs.uk/publication/next-steps-on-the-nhs-five-year-forward-view/. [last accessed 25 March 2020].

25. Grist R, Porter J, Stallard P. Mental Health Mobile Apps for Preadolescents and Adolescents: A Systematic Review. Journal of Medical Internet Research. 2017;19(5). doi:10.2196/jmir.7332.

26. Office of Communications, 2020. Children and parents: Media use and attitudes report 2019 [online]. Available from: https://www.ofcom.org.uk/__data/assets/pdf_file/0023/190616/children-media-use-attitudes-2019-report.pdf. [last accessed 16 April 2020].

27. Melia R, Francis K, Hickey E, et al. Mobile Health Technology Interventions for Suicide Prevention: Systematic Review. JMIR Publications. 2020;8(1).

For peer review only

28. Rasing S P A, Stikkelbroek Y A J, Bodden D H M. Is Digital Treatment the Holy Grail? Literature Review on Computerized and Blended Treatment for Depressive Disorders in Youth. International Journal of Environmental Research and Public Health. 2020;17(153).

29. Melia R, Francis K, Hickey E, Bogue J, Duggan J, O'Sullivan M, Young K. Mobile health technology interventions for suicide prevention: Systematic review. JMIR mHealth and uHealth. 2020;8(1):e12516

30. Forte A, Sarli G, Polidori L, Lester D, Pompili M. The role of new technologies to prevent suicide in adolescence: a systematic review of the literature. Medicina. 2021 Feb;57(2):109.

31. Cliffe B, Tingley J, Greenhalgh I, Stallard P. Mhealth interventions for self-harm: scoping review. Journal of medical internet research. 2021 Apr 30;23(4):e25140.

32. Stallard P, Porter J, Grist R. Safety, acceptability, and use of a smartphone app, BlueIce, for young people who self-harm: protocol for an open phase I trial. JMIR research protocols. 2016;5(4), e217.

33. Stallard P, Porter J, Grist R. A smartphone app (BlueIce) for young people who self-harm: open phase 1 pre-post trial. JMIR mHealth and uHealth. 2018;6(1), e32.

34. NHS Digital Developer, 2019. Developer.nhs.uk. Digital Assessment Questions – Beta [online]. Available from: https://developer.nhs.uk/digital-tools/daq/. [last accessed 13 April 2020]

35. Vrouva I, Fonagy P, Fearon P, et al. The Risk-Taking and Self-Harm Inventory for Adolescents (RTSHIA): Development and Psychometric Evaluation. Psychological Assessment. 2010;22(4):852-865. doi:10.1037/a0020583.

36. Kidger J, Heron J, Lewis G, et al. Adolescent self-harm and suicidal thoughts in the ALSPAC cohort: a self-report survey in England. BMC Psychiatry. 2012;12(69). https://doi.org/10.1186/1471-244X-12-69

37. Costello E J, Angold A. Scales to Assess Child and Adolescent Depression: Checklists, Screens, and Nets. Journal of the American Academy of Child & Adolescent Psychiatry. 1988;27(6): 726-737.

38. Beck A T, Weissman A, Lester D, et al. The measurement of pessimism: the hopelessness scale. Journal of consulting and clinical psychology. 1974;42(6):861-865.

39. Kazdin A E, French N H, Unis A S, et al. Hopelessness, depression, and suicidal intent among psychiatrically disturbed inpatient children. Journal of Consulting and Clinical Psychology. 1983;51(4):504-510. https://doi.org/10.1037/0022-006X.51.4.504

40. Chorpita B F, Yim L, Moffitt C, et al. Assessment of symptoms of DSM-IV anxiety and depression in children: a revised child anxiety and depression Scale. Behavior Research and Therapy. 2000;38:835–855. doi: 10.1016/S0005-7967(99)00130-8.

41. Goodman R. The Strengths and Difficulties Questionnaire: A Research Note. Journal of Child Psychology and Psychiatry. 1997;38(5):581-586.

42. Espie C A, Kyle S D, Hames P, et al. The Sleep Condition Indicator: a clinical screening tool to evaluate insomnia disorder. BMJ open. 2014;4(3). e004183.

For peer review only

For peer review only - http://bmjopen.bmj.com/site/about/guidelines.xhtml

43. Grist R, Porter J, Stallard P. Acceptability, Use, and Safety of a Mobile Phone App (BlueIce) for Young People Who Self-Harm: Qualitative Study of Service Users' Experience. JMIR mental health. 2018;5(1).

44. Stevens K J. Assessing the performance of a new generic measure of health related quality of life for children and refining it for use in health state valuation. Applied Health Economics and Health Policy. 2011; 9(3): 157-169.

45. Ratcliffe J, Stevens K, Flynn T,et al. An assessment of the construct validity of the CHU9D in the Australian adolescent general population. Quality of Life Research. 2012;21(4):717-25.

46. Furber G, Segal L. The validity of the Child Health Utility instrument (CHU9D) as a routine outcome measure for use in child and adolescent mental health services. Health and quality of life outcomes. 2015;13(1):1.

47. Drummond M F, Sculpher M J, Claxton K, et al. Methods for the economic evaluation of health care programmes. Oxford: Oxford university press. 2015.

48. Husereau D, Drummond M, Petrou S, et al. Consolidated health economic evaluation reporting standards (CHEERS) statement. BMJ. 2013;346:f1049.

49. Briggs A, Fenn P. Confidence intervals or surfaces? Uncertainty on the cost-effectiveness plane. Health economics.1998;7(8):723-740.

50. Fenwick E, Byford S. A guide to cost-effectiveness acceptability curves. BJ Psych. 2005;187(2):106-108.

51. Faria R, Gomes M, Epstein D, et al. A guide to handling missing data in cost-effectiveness analysis conducted within randomised controlled trials. Pharmacoeconomics. 2014;32(12):1157-1170.

52. Schulz K F, Altman D G, Moher D, for the CONSORT Group. CONSORT 2010 Statement: updated guidelines for reporting parallel group randomised trials. Lancet. 2010;375(9721):1136.

For peer review only

For peer review only - http://bmjopen.bmj.com/site/about/guidelines.xhtml

SPIRIT

STANDARD PROTOCOL ITEMS: RECOMMENDATIONS FOR INTERVENTIONAL TRIALS

SPIRIT 2013 Checklist: Recommended items to address in a clinical trial protocol and related documents*

Section/item	Item No	Description
**Administrative information**		
Title	1	Beating Adolescent Self harm (BASH). A randomised controlled trial comparing usual care versus usual care plus a smartphone self-harm prevention app (BlueIce) in young adolescents aged 12-17 who self-harm: study protocol (Paper page 1)
Trial registration	2a	ISRCTN registry, registration number ISRCTN 10541045. Registered on 22/07/2019 (Paper page 2)
	2b	All items from the World Health Organization Trial Registration Data Set (N/A)
Protocol version	3	Attached protocol, dated 18.01.2019, version 1.
Funding	4	This study was funded by the National Institute for Health Research, Research for Patient Benefit programme (PB-PG-1217-20004). (Paper page 13)
Roles and responsibilities	5a	PS, GT, AML and SR designed and obtained funding for the study. PS, IG and JT drafted the study protocol. All authors contributed to and approved the final version (Paper page 13)
	5b	Oxford Health NHS Foundation Trust (Paper page 13l)
	5c	The sponsor/funder will have no role in interpreting data, writing reports or decisions to publish findings (Paper page 13).
	5d	Trial management defined (Paper page11)
**Introduction**		
Background and rationale	6a	Paper page 3-4
	6b	This is a two arm, single blind, randomised controlled trial (RCT) comparing the addition of the BlueIce self-help app to usual face-to-face specialist mental health care (UC+BI) with usual specialist mental health care (UC). (Paper page 5)

For peer review only

Objectives	7	The aims of this trial are to determine the effectiveness, cost-effectiveness and acceptability of adding BlueIce to usual face-to-face specialist mental health care in the reduction of self-harm in adolescents. (Paper page 4)
Trial design	8	Paper page 5 trial design

Methods: Participants, interventions, and outcomes

Study setting	9	Paper page 5 setting and participants
Eligibility criteria	10	Paper page 5 under setting and participants
Interventions	11a	Paper pages 6-7 under interventions
	11b	N/a
	11c	N/a
	11d	Paper page 6 usual care.
Outcomes	12	Outcomes defined paper pages 8-9.
Participant timeline	13	Assessment timeline defined paper pages 7-8.
Sample size	14	Sample size defined paper page 9.
Recruitment	15	N/a

Methods: Assignment of interventions (for controlled trials)

Allocation:		
Sequence generation	16a	Randomisation defined paper page 5-6
Allocation concealment mechanism	16b	Concealment described paper page 6
Implementation	16c	Participant allocation described paper page 6
Blinding (masking)	17a	Blinding described paper page 6
	17b	N/a

Methods: Data collection, management, and analysis

Data collection methods	18a	Outcome assessments and assessment schedule defined paper pages 7-9

	18b	N/a
Data management	19	Data management procedures are described in the attached protocol page 14.
Statistical methods	20a	Described in statistical analysis paper page 10
	20b	Described in statistical analysis paper page 10
	20c	Described in statistical analysis paper page 10
**Methods: Monitoring**		
Data monitoring	21a	No interim analysis was planned and as such a DMC was not considered necessary. Data monitoring and review of adverse events was undertaken by the SSG. Trial management section paper page 11
	21b	N/a. No interim analysis was planned.
Harms	22	Reporting of serious adverse events defined in attached protocol page 13.
Auditing	23	N/a
**Ethics and dissemination**		
Research ethics approval	24	Ethical approval stated paper page 11
Protocol amendments	25	Protocol will be updated with version number and date as appropriate
Consent or assent	26a	Summarised paper page 5
	26b	N/a
Confidentiality	27	Management of personal information described in the attached protocol page 14.
Declaration of interests	28	Competing interest statement declared paper page 13.
Access to data	29	Summarised in attached protocol page 14
Ancillary and post-trial care	30	N/a
Dissemination policy	31a	Dissemination plans described in paper page 11.
	31b	Not specified
	31c	None made

review only

For peer review only - http://bmjopen.bmj.com/site/about/guidelines.xhtml

Appendices

Informed consent materials	32	Consent forms uploaded
Biological specimens	33	N/a

*It is strongly recommended that this checklist be read in conjunction with the SPIRIT 2013 Explanation & Elaboration for important clarification on the items. Amendments to the protocol should be tracked and dated. The SPIRIT checklist is copyrighted by the SPIRIT Group under the Creative Commons "Attribution-NonCommercial-NoDerivs 3.0 Unported" license.

For peer review only
